# Vaccines for post-exposure prophylaxis against varicella (chickenpox) in children and adults

**DOI:** 10.1590/S1516-31802012000200014

**Published:** 2012-04-03

**Authors:** Kristine Macartney, Peter McIntyre

## Abstract

**BACKGROUND:**

Live attenuated varicella vaccines for the prevention of varicella (chickenpox) has been demonstrated both in randomised controlled trials (RCTs) and in population-based immunisation programmes in countries such as the United States. However, many countries do not routinely immunise children against varicella, and exposures continue to occur. Although the disease is often mild, complications such as secondary bacterial infection, pneumonitis and encephalitis occur in about 1% of cases, usually leading to hospitalisation. The use of varicella vaccine in persons who have recently been exposed to the varicella zoster virus has been studied as a form of post-exposure prophylaxis (PEP).

**OBJECTIVE:**

To assess the efficacy and safety of vaccines for use as PEP for the prevention of varicella in children and adults.

**CRITERIA FOR CONSIDERING STUDIES FOR THIS REVIEW:**

We searched the Cochrane Central Register of Controlled Trials (CENTRAL) (The Cochrane Library, 2008, Issue 1); MEDLINE (1966 to February 2008); and EMBASE (January 1990 to February 2008).

**SELECTION CRITERIA:**

RCTs and quasi-RCTs of varicella vaccine for PEP compared with placebo or no intervention. The outcome measures were efficacy in prevention of clinical cases and/or laboratory-confirmed clinical cases and adverse effects following vaccination.

**DATA COLLECTION AND ANALYSIS:**

Two review authors independently extracted and analysed data using Review Manager software.

**MAIN RESULTS:**

Three studies involving 110 healthy children who were siblings of household contacts were identified as suitable for inclusion. The studies varied in quality, study design, vaccine used, and outcomes measured and, as such, were not suitable for meta-analysis. Overall, 13 out of 56 vaccine recipients (18%) developed varicella compared with 42 out of 54 placebo (or no vaccine) recipients (78%). Of the vaccine recipients who developed varicella, the majority only had mild disease (with less than 50 skin lesions). In the three studies, most subjects received PEP within three days following exposure; too few subjects were vaccinated four to five days post exposure to ascertain the efficacy of vaccine given more than three days after exposure. No included studies reported on adverse events following immunisation.

**AUTHORS CONCLUSIONS:**

These small trials suggest varicella vaccine administered within three days to children following household contact with a varicella case reduces infection rates and severity of cases. No RCTs for adolescents or adults were identified. However safety was not adequately addressed.

This is the abstract of a Cochrane Review published in the Cochrane Database of Systematic Reviews (cdsr) 2011, issue 08, doi: 10.1002/14651858.CD001833.pub3 (http://www.thecochranelibrary.com). For full citation and authors details, see reference 1.

For Latin America and the Caribbean, the full text is freely available from: http://cochrane.bvsalud.org/cochrane/show.php?db=reviews&mfn=932&id=CD001833&lang=pt&dblang=&lib=COC&print=yes
